# Correction: Imputation of the Rare *HOXB13* G84E Mutation and Cancer Risk in a Large Population-Based Cohort

**DOI:** 10.1371/journal.pgen.1005114

**Published:** 2015-04-14

**Authors:** The PLOS Genetics Staff

In the funding statement, the sentence “This work was also supported by the UCSF Program in Prostate Cancer Translational Biology” is incorrect. The correct sentence is: “This work was also supported by the Goldberg-Benioff Program in Cancer Translational Biology.”

There are errors in the abstract. The sentence “Imputation authenticity was confirmed via a novel classification and regression tree method, and then empirically validated analyzing a subset of these subjects plus an additional 1,789 men from the California Men’s Health Study specifically genotyped for the G84E mutation (r^2^ = 0.57, 95% CI = 0.37–0.77)” is incorrect. The correct sentence is: “Imputation authenticity was confirmed via a novel classification and regression tree method, and then empirically validated analyzing a subset of these subjects plus an additional 1,789 men from Kaiser specifically genotyped for the G84E mutation (r^2^ = 0.57, 95% CI = 0.37–0.77).”

The sentence “The age-specific risk of prostate cancer among G84E mutation carriers was higher than among non-carriers, and this difference increased with age.” in the abstract is incorrect. The correct sentence is: “The age-specific risk of prostate cancer among G84E mutation carriers was higher than among non-carriers.”

The last sentence in the abstract is incorrect. The correct sentence is “The G84E mutation was also associated with an increase in risk for the fourteen other most common cancers considered collectively (p = 5.8x10^-4^) and more so in cases diagnosed with multiple cancer types, both those including and not including prostate cancer, strongly suggesting pleiotropic effects.”

A full, corrected version of the abstract is below.

“An efficient approach to characterizing the disease burden of rare genetic variants is to impute them into large well-phenotyped cohorts with existing genome-wide genotype data using large sequenced referenced panels. The success of this approach hinges on the accuracy of rare variant imputation, which remains controversial. For example, a recent study suggested that one cannot adequately impute the *HOXB13* G84E mutation associated with prostate cancer risk (carrier frequency of 0.0034 in European ancestry participants in the 1000 Genomes Project). We show that by utilizing the 1000 Genomes Project data plus an enriched reference panel of mutation carriers we were able to accurately impute the G84E mutation into a large cohort of 83,285 non-Hispanic White participants from the Kaiser Permanente Research Program on Genes, Environment and Health Genetic Epidemiology Research on Adult Health and Aging cohort. Imputation authenticity was confirmed via a novel classification and regression tree method, and then empirically validated analyzing a subset of these subjects plus an additional 1,789 men from Kaiser specifically genotyped for the G84E mutation (r^2^ = 0.57, 95% CI = 0.37–0.77). We then show the value of this approach by using the imputed data to investigate the impact of the G84E mutation on age-specific prostate cancer risk and on risk of fourteen other cancers in the cohort. The age-specific risk of prostate cancer among G84E mutation carriers was higher than among non-carriers. Risk estimates from Kaplan-Meier curves were 36.7% versus 13.6% by age 72, and 64.2% versus 24.2% by age 80, for G84E mutation carriers and non-carriers, respectively (p = 3.4x10^-12^). The G84E mutation was also associated with an increase in risk for the fourteen other most common cancers considered collectively (p = 5.8x10^-4^) and more so in cases diagnosed with multiple cancer types, both those including and not including prostate cancer, strongly suggesting pleiotropic effects.”
