# Correction: Imputation of the Rare *HOXB13* G84E Mutation and Cancer Risk in a Large Population-Based Cohort

**DOI:** 10.1371/journal.pgen.1005362

**Published:** 2015-06-26

**Authors:** The PLOS Genetics Staff

## Notice of Republication

This article was republished on June 08, 2015, to correct errors in the funding information and the abstract. This republication addresses the issues noted in the correction published on April 14, 2015. Please download this article again to view the correct version. The originally published, uncorrected article and the republished, corrected article are provided here for reference.

## Supporting Information

S1 File Originally published, uncorrected article. (PDF)

S2 File Republished, corrected article. (PDF)
