# Supplementary material for: Correction: Imputation of the Rare HOXB13 G84E Mutation and Cancer Risk in a Large Population-Based Cohort
Source: PLoS Genet. 2015 Jun 26;11(6):e1005362. doi: 10.1371/journal.pgen.1005362 (PMC4483271; doi:10.1371/journal.pgen.1005362)
Supplement: S1 File — (PDF) [file pgen.1005362.s001.pdf]

PLOS | GENETICS

RESEARCH ARTICLE

Imputation of the Rare *HOXB13* G84E Mutation and Cancer Risk in a Large Population-Based Cohort

Thomas J. Hoffmann[1,2], Lori C. Sakoda[3], Ling Shen[3], Eric Jorgenson[3], Laurel A. Habel[3], Jinghua Liu[2], Mark N. Kvale[2], Maryam M. Asgari[3], Yambazi Banda[2], Douglas Corley[3], Lawrence H. Kushi[3], Charles P. Quesenberry Jr.[3], Catherine Schaefer[3], Stephen K. Van Den Eeden[3,4], Neil Risch[1,2,3], John S. Witte[1,2,4]*

1 Department of Epidemiology and Biostatistics, University of California San Francisco, San Francisco, California, United States of America, **2** Institute for Human Genetics, University of California San Francisco, San Francisco, California, United States of America, **3** Division of Research, Kaiser Permanente, Northern California, Oakland, California, United States of America, **4** Department of Urology, University of California San Francisco, San Francisco, California, United States of America

* wittej@humgen.ucsf.edu

Abstract

An efficient approach to characterizing the disease burden of rare genetic variants is to impute them into large well-phenotyped cohorts with existing genome-wide genotype data using large sequenced referenced panels. The success of this approach hinges on the accuracy of rare variant imputation, which remains controversial. For example, a recent study suggested that one cannot adequately impute the *HOXB13* G84E mutation associated with prostate cancer risk (carrier frequency of 0.0034 in European ancestry participants in the 1000 Genomes Project). We show here that—by utilizing the 1000 Genomes Project data plus an enriched reference panel of mutation carriers—we were able to accurately impute the G84E mutation into a large cohort of 83,285 non-Hispanic White participants from the Kaiser Permanente Research Program on Genes, Environment and Health Genetic Epidemiology Research on Adult Health and Aging cohort. Imputation authenticity was confirmed via a novel classification and regression tree method, and then empirically validated analyzing a subset of these subjects plus an additional 1,789 men from the California Men's Health Study specifically genotyped for the G84E mutation (r2 = 0.57, 95% CI = 0.37–0.77). We then show the value of this approach by using the imputed data to investigate the impact of the G84E mutation on age-specific prostate cancer risk and on risk of fourteen other cancers in the cohort. The age-specific risk of prostate cancer among G84E mutation carriers was higher than among non-carriers, and this difference increased with age. Risk estimates from Kaplan-Meier curves were 36.7% versus 13.6% by age 72, and 64.2% versus 24.2% by age 80, for G84E mutation carriers and non-carriers, respectively (p = 3.4×10−12). The G84E mutation was also suggestively associated with an increase in risk for the following cancer sites by approximately 50% in a pleiotropic manner: breast, non-Hodgkin's lymphoma, kidney, bladder, melanoma, endometrium, and pancreas (p = 0.042).

OPEN ACCESS

Citation: Hoffmann TJ, Sakoda LC, Shen L, Jorgenson E, Habel LA, Liu J, et al. (2015) Imputation of the Rare *HOXB13* G84E Mutation and Cancer Risk in a Large Population-Based Cohort. PLoS Genet 11(1): e1004930. doi:10.1371/journal.pgen.1004930

Editor: Peter M. Visscher, Queensland Institute of Medical Research, AUSTRALIA

Received: June 2, 2014

Accepted: December 1, 2014

Published: January 28, 2015

Copyright: © 2015 Hoffmann et al. This is an open access article distributed under the terms of the Creative Commons Attribution License, which permits unrestricted use, distribution, and reproduction in any medium, provided the original author and source are credited.

Data Availability Statement: Over 78% of GERA participants consented to having their data shared with dbGaP (accession number phs000674.v1.p1), where the data are now available. Data are also available via the RPGEH portal: https://rpgehportal.kaiser.org.

Funding: This work was supported by grants R21 AG046616 and K01 DC013300 to TJH, and U01 CA127298 and R01 CA088164 to JSW from the National Institutes of Health. This work was also supported by the UCSF Program in Prostate Cancer Translational Biology. Support for participant

enrollment, survey completion, and biospecimen collection for the RPGEH was provided by the Robert Wood Johnson Foundation, the Wayne and Gladys Valley Foundation, the Ellison Medical Foundation, and Kaiser Permanente national and regional community benefit programs. Genotyping of the GERA cohort was funded by a grant from the National Institute on Aging, National Institute of Mental Health, and the National Institute of Health Common Fund (RC2 AG036607 to CAS and NJR). The funders had no role in study design, data collection and analysis, decision to publish, or preparation of the manuscript.

Competing Interests: The authors have declared that no competing interests exist.

Author Summary

An efficient approach to characterizing the disease burden of rare genetic variants is to impute them into existing well-phenotyped cohorts with genome-wide data by using large sequenced reference panels; however, the efficacy of this approach remains controversial. A recent study suggested that it is not possible to impute the rare *HOXB13* G84E variant using neighboring SNP markers. We show that by using an enriched reference sequenced sample of 22 mutation carriers, we were able to impute this mutation into a large cohort of 83,285 non-Hispanic White individuals from the Kaiser Permanente Research Program on Genes, Environment, and Health Genetic Epidemiology Research on Adult Health and Aging (GERA) cohort. The imputation was confirmed via a novel classification and regression tree method, and then empirically validated by direct mutation genotyping of a subset of 1,673 of these individuals in addition to 1,789 other men from Kaiser. Using the same GERA cohort, we then confirmed that the G84E mutation is associated with increased risk of prostate cancer, and estimated the age-specific risk for carriers of the mutation. Finally, we obtained evidence that the mutation is associated with additional types of cancer in the GERA cohort.

Introduction

The impact of rare genetic variants on human diseases and traits is of great current interest. A number of rare variants have been implicated in common diseases, and some genome-wide association study signals may reflect linkage disequilibrium with underlying rare variants [1]. However, it has remained challenging to obtain adequate power to analyze rare variants in population-based studies that are not enriched for the variant—as opposed to potentially enriched family-based studies—unless the study sample size is very large [2]. In addition, many genome-wide association studies (GWAS) have not included rare variants in their analysis [3,4].

Instead of assaying rare variants directly, a more efficient approach may be to use existing sequencing data as a reference panel with which to impute rare variants, although it remains controversial as to how effective this approach will be, as well as the minimum allele frequency that will be accessible by this approach. While some argue that it is not feasible to impute rare variants with MAF < 0.03 [5], recent work indicates that it is possible to impute not only variants with minor allele frequency (MAF) between 0.01 and 0.05, but even variants with MAF < 0.01 from GWAS data [6–16].

One example of such a rare variant is the transcription factor homeobox 13 (*HOXB13*) mutation G84E (rs138213197; chromosome 17; build 37 position 46,805,705), which has been associated with a high risk of prostate cancer and replicated in at least ten case-control studies [17–30], with a recent meta-analysis odds ratio (OR) of 4.51 [22]. Most previous work has been based on case-control samples, but a few groups have also looked at the lifetime risk [24,27]. In addition to prostate cancer, the *HOXB13* gene may be involved in the development of ovarian cancer [31], bladder cancer progression [32], oral squamous cell carcinoma aggressiveness [33], and breast cancer aggressiveness and tamoxifen treatment response [34]. However, results for the effect of the G84E mutation on breast or colorectal cancer susceptibility are mixed [26,35,36]. Taken together, evidence supports further exploration of a potential pleiotropic effect of the G84E mutation on multiple cancers.

One group has reported that the G84E mutation could not be imputed using the SNPs included on the custom Illumina Collaborative Oncological Gene-Environment Study (iCOGS)

array, though they did identify experimental evidence for a synthetic association of more common genetic variants in the region with HOXB13 G84E [37]. However, another group has found an ancestral haplotype containing the mutation, suggesting that imputation should be possible [19].

We show here that imputation of the G84E mutation is possible by using a mutation carrier enriched reference panel and applying the imputation to a large cohort of 83,285 non-Hispanic White individuals with genome-wide genotyping data from the multi-ethnic Kaiser Permanente (KP) Research Program on Genes, Environment, and Health (RPGEH) Genetic Epidemiology Research on Aging (GERA) cohort. To assess the accuracy of the imputation, we applied an easily implemented classification and regression tree (CART) method to phased haplotypes to identify the founder haplotype carrying the G84E mutation. We also confirmed the founder haplotype via standard phasing and multi-marker correlation approaches [38]. We subsequently validated the imputation by genotyping the G84E variant in a subset of 1,673 RPGEH GERA individuals who also had genome-wide genotype data, in addition to 1,789 men who also had genome-wide genotype data from the RPGEH and from the Kaiser California Men's Health Study (CMHS) [39] (used only for the empirical validation), and compared the direct genotype results to the imputed values. Finally, we used the imputed data to estimate the lifetime risk of prostate cancer among mutation carriers and non-carriers and to evaluate the association between G84E and risk of the other 14 most common cancers in the RPGEH GERA cohort.

Results

Imputation results

Imputation of the G84E mutation into the 83,285 non-Hispanic Whites in the RPGEH GERA cohort using the enriched reference panel led to a high level of confidence in the imputation ($r_{info}^2 = 0.96$). Calculation of r^2 by LOOCV based on the reference panel gave an estimated $r^2 = 0.92$ (24 correctly predicted mutation bearing haplotypes; 2,345 correctly predicted non-bearing haplotypes; one predicted mutation carrier that was not, and no predicted non-carriers that were carriers).

Using several methods, we confirmed the ability to impute the G84E mutation. The haplotype CART approach, a prediction tree built from the computationally phased enriched reference panel, is shown in the first tree in black in Fig. 1. The first split occurs with the T allele at the best pairwise tag SNP rs145922598 ($r^2 = 0.12$; no SNP has a strong pairwise correlation because it is a rare founder mutation). Following the T variant, the next split is at the A allele of SNP rs75947881, followed by the A allele of SNP rs8067245, which captures the majority of the rest of the variation and correctly predicts all 24 mutation carriers but also 2 individuals as mutation carriers that are not; no mutation carriers are incorrectly predicted as non-carriers. Any further splits would likely be overtraining; standard CART procedures can be used to prevent this [40], although the best end point may be difficult to determine when there are few carriers. The correlation of the mutation carrier and non-carrier genotypes derived from this tree for the entire RPGEH GERA dataset with those obtained from the imputation is moderately high ($r^2 = 0.63$, 95% CI = 0.60–0.66). Thus, the founder haplotype can be reasonably captured just from these three SNPs. Further, when we removed these three SNPs found by the CART approach and repeated the CART analysis on the remaining SNPs, we found an additional prediction tree of four SNPs shown in the second tree in green in Fig. 1. The splits leading to the final tree are at rs8073596 = T, rs62065362 = T, rs4793974 = T, and rs281613067 = A. In this case, 20 mutation carriers are correctly identified, whereas 4 carriers are predicted to be non-carriers and 3 non-carriers are predicted to be carriers. This second tree had a high correlation

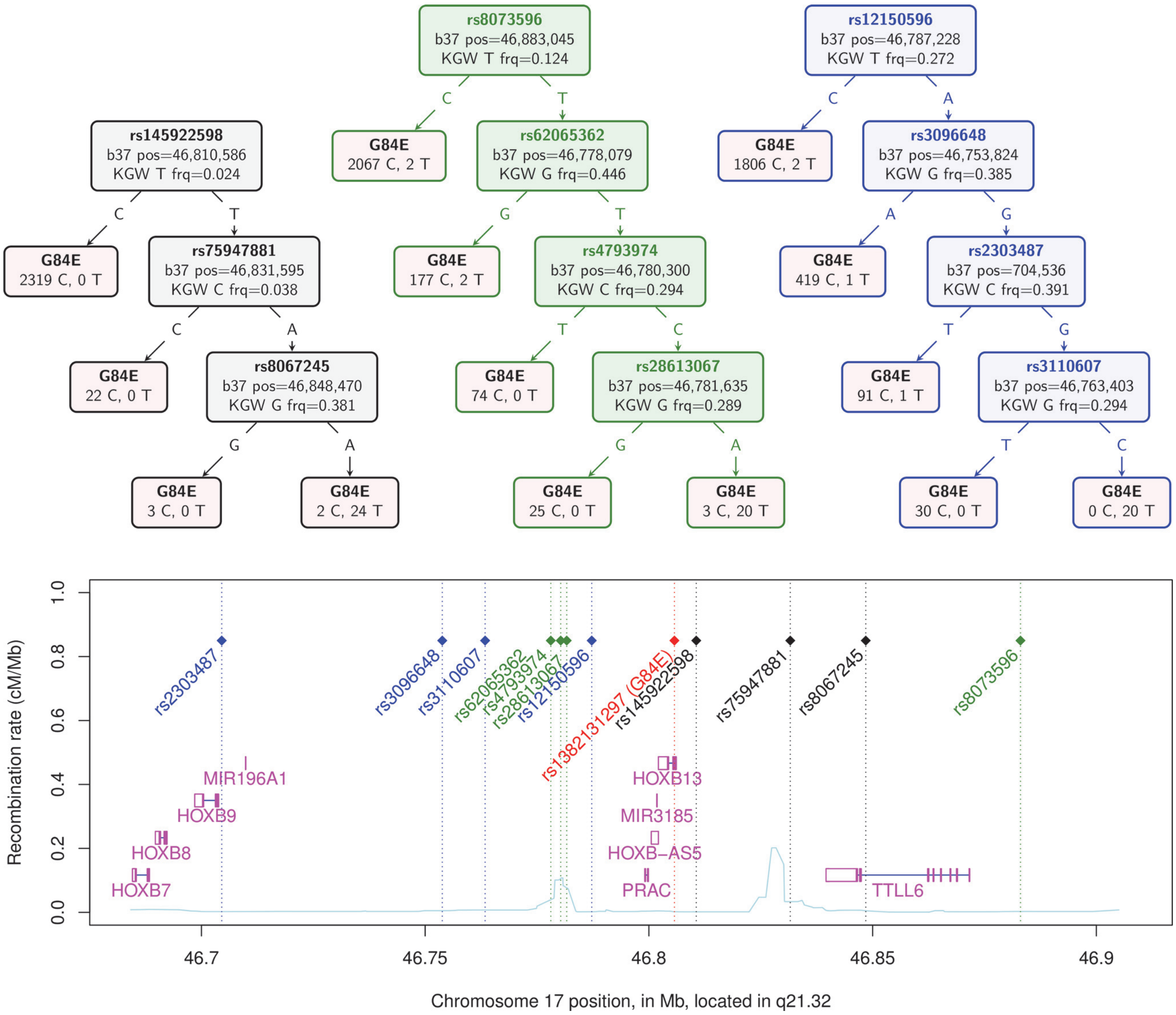

Figure 1. Confirmation of *HOXB13* G84E mutation status from classification and regression tree. The top of the figure shows three CART trees produced for the computationally phased haplotypes of the enriched reference panel of 93 individuals (22 carriers) plus 1000 Genomes data (2 carriers). Listed in the trees are the splits that classify the G84E mutation. The leaves in the tree contain the best guess classification of G84E on the top, and the number of reference alleles on the left and the number of G84E mutations on the right. The first tree, in black, is formed from selecting amongst all 57 SNPs +/− 3 crossovers. The second tree, in green, is formed from selecting from the same set of SNPs except excluding the 3 found in the first tree. The third tree, in blue, is formed from selecting amongst the same set of SNPs except excluding the 7 found in the first and second trees. Below the trees is a local chromosome plot of the region in reference to the surrounding genes and recombination rate of the region, with the color of the rs# for each SNP indicating the tree from which it was derived. KGW, 1000 Genomes white race/ethnicity individuals; frq, frequency.

doi:10.1371/journal.pgen.1004930.g001

with the original imputed genotype of $r^2 = 0.73$ (95% CI = 0.69–0.76). We conducted the process one more time by removing all 7 SNPs found in the first two trees, and repeating the CART analysis, with results shown in the third tree in blue in Fig. 1. In this case, 20 mutation carriers were correctly called, while 4 mutation carriers were incorrectly called non-carriers; no individuals predicted to be carriers did not carry the mutation. This third tree had a correlation with the original imputed genotype of $r^2 = 0.67$ (95% CI = 0.64–0.71). These results indicate that prediction of mutation carriers does not hinge on just a few sentinel SNPs in the region. In addition, repeating the original imputation analysis but only including the 11 SNPs in these

three trees yielded an $r_{info}^2 = 0.92$, and a correlation with the original imputed genotype of $r^2 = 0.93$, 95% CI = 0.91–0.94. While the original imputation analysis used all 57 SNPs in the region, this analysis also shows that a much reduced set (e.g., the 11 SNPs described above) can capture most of the information needed to predict mutation carrier status by imputation.

For our final computational approach, we calculated the multi-marker correlation of the G84E mutation using an alternative haplotype estimation procedure provided in PLINK [38]. Using the three SNPs rs145922598, rs75947881, and rs8067245 gave $r^2 = 0.82$. Not including these three SNPs, we found a set of 4 SNPs that gave $r^2 = 0.69$.

Comparison of imputed to genotyped mutation carriers. As a final confirmation of the imputation, we genotyped the G84E mutation on a subset of 3,462 White men who also had Axiom genotype data. Fig. 2 shows the genotyping cluster plot of the mutation; there are two well-separated clusters. We highlight the different categories of individuals based on whether they were directly genotyped as carriers versus imputed to be carriers based on the most likely or "best guess" genotype. There are no true genotyped mutation carriers that were not also imputed carriers; however, only 15/26 individuals who were predicted by imputation to be carriers were actually genotyped as carriers. We obtained a lower imputation $r^2 = 0.57$ (95% CI = 0.37–0.77) than we previously estimated by the various computational methods.

The discrepancy between the original imputation r_{info}^2 and the experimentally validated r^2 above is due to the extreme enrichment of mutation carriers in the original imputation panel, leading to a different proportion of haplotype carriers that are true mutation carriers in our genotyped dataset than in the reference panel. The original imputation panel consisted of 24 directly sequenced mutation carriers and 448 White non-carriers (and all 713 of other races/ethnicities, see Methods). Because the ancestral haplotype (without the mutation) is also uncommon, only two of the 448 White non-carriers had the ancestral haplotype (and none of those of other races/ethnicities). Thus, the extreme oversampling of mutation carriers led to an inflated r-square. To verify this, we reversed the process and used the genotyped validation sample as the reference to impute G84E into the original enriched reference panel. If the proportion of haplotype carriers that are true mutation carriers is higher in the original reference panel than in the validation subset, then even using the validation sample as the reference would still predict a high rate of mutation carriers among haplotype carriers in the original reference panel. In this reversed process, we observed $r_{info}^2 = 0.95$ and a correlation of imputed genotypes to the directly observed genotypes (for the mutation) of $r^2 = 0.87$ and correctly identified 22/24 mutation carriers and one incorrectly declared carrier. By design, the proportion of ancestral haplotype carriers in the original enriched reference sample is higher than what we observed in the validation sample, indicating that the original imputation algorithm was valid.

To further quantify the effect of the over-sampling that took place in the creation of the original enriched reference sample and show the estimates are actually consistent, we can downward adjust the original r^2 estimate to account for mutation over-sampling in the original reference. When we adjust the original r^2 estimate for oversampling, we get an estimate of $r^2 = 0.70$ (see Methods). This is higher than what was found on the genotyped array, but within the 95% CI of the estimate (see Methods, and also note that this adjustment was based on very small sample sizes and hence also subject to statistical fluctuation).

G84E Ancestry

We show a smoothed estimate of the G84E mutation carrier frequency using the expected additive coding of each individual's imputed genotype and adjusting for incomplete LD in Fig. 3,

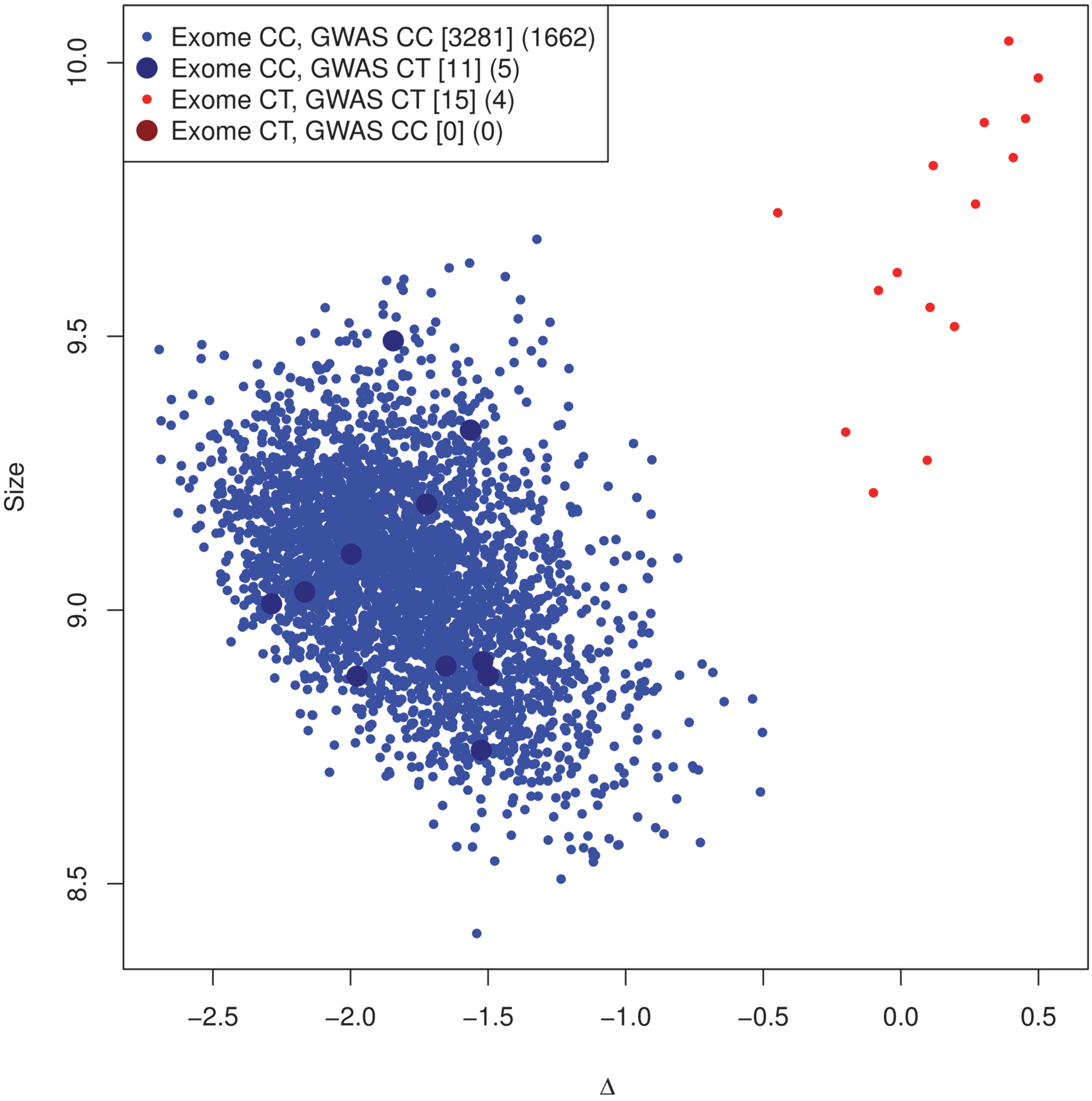

Figure 2. Genotyping cluster plot of the G84E variant. A subset of the RPGEH GERA cohort, in addition to the CMHS cohort, were additionally genotyped at the G84E variant. All carriers are imputed correctly, but some individuals are falsely identified as carriers ($r^2 = 0.57$, 95% CI = 0.37–0.77). This is because of lack of specificity of the ancestral haplotype for mutation carriers. Counts of (Exome array genotype call, GWAS imputation call) categories for RPGEH GERA and Men's Health cohort are given in brackets [.], and for RPGEH GERA alone in parenthesis (.). The most likely/best guess genotypes are given for the imputed data. Discordances are noted with the larger points.

doi:10.1371/journal.pgen.1004930.g002

with individuals with >25% Ashkenazi ancestry excluded, and with the Human Genome Diversity Project [41] groups overlaid to enhance interpretability. The mutation is most prevalent in northwestern European and Russian groups (approximately 0.8% vs. 0.4% overall). By contrast, the carrier frequency was much lower (approximately 0.15%) in Southern Europeans and individuals of Ashkenazi ancestry.

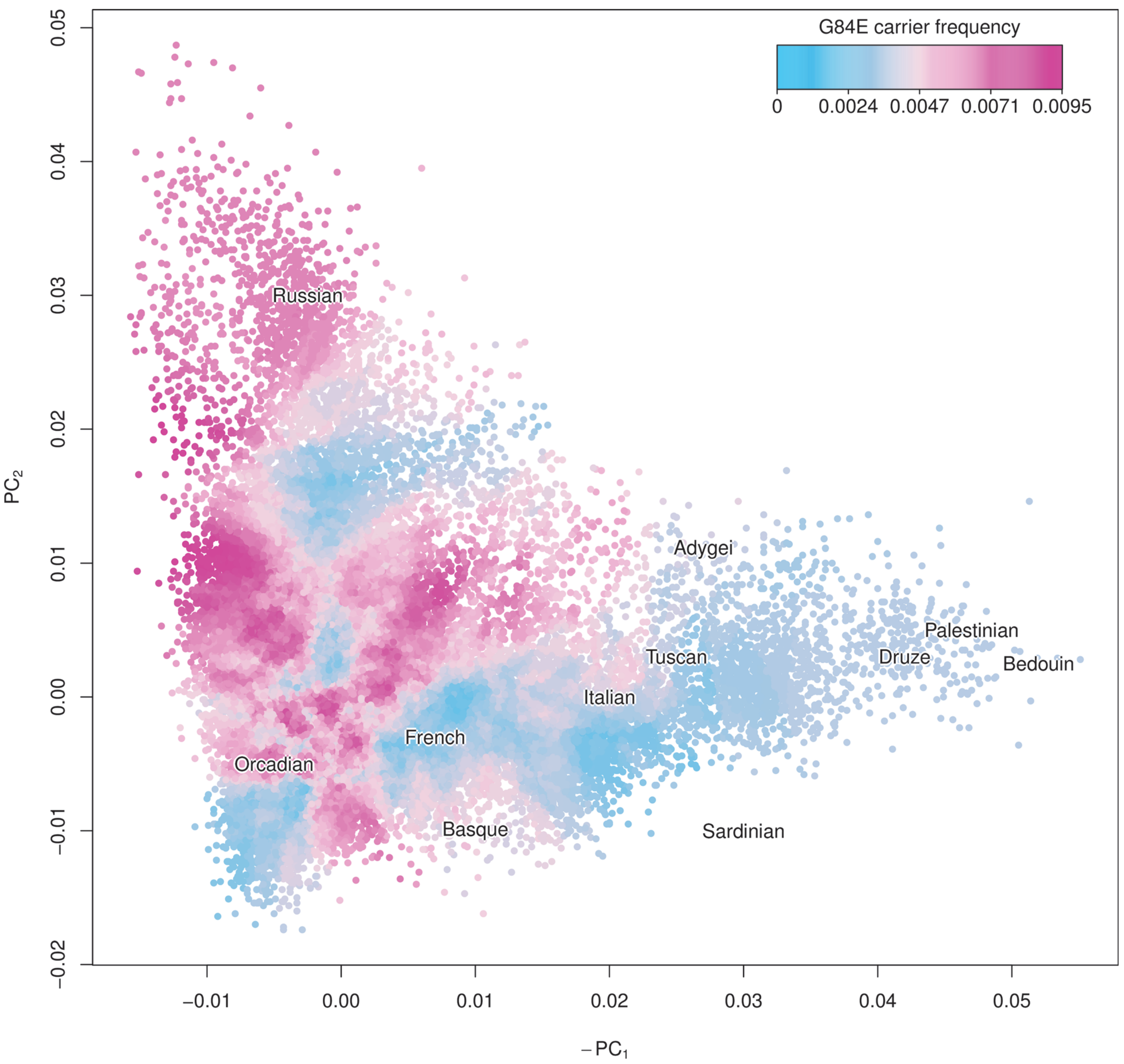

Figure 3. Ancestry of the HOXB13 G84E variant. Using the first two principal components (PCs) we created a smoothed estimate of the carrier frequency of each individual's expected additive coding by using the 2,000 closest individuals (Euclidean distance) to calculate a G84E carrier frequency at that location, excluding individuals with >25% Ashkenazi ancestry. Text for the center of each Human Genome Diversity Project (HGDP) population is given to enhance interpretation; the mutation is most prevalent in northwestern Europe and Russian groups. To further adjust for incomplete LD, we multiplied the imputation carrier frequency by the r^2 estimate of 0.57.

doi:10.1371/journal.pgen.1004930.g003

Application to a large cohort

After exclusions of first-degree relatives and others in identifying all cancer cases and controls (described in the Methods section), a total of 74,625 individuals from RPGEH GERA were used to evaluate the association between the *HOXB13* G84E mutation and cancer.

G84E and prostate cancer risk. Focusing first on prostate cancer (3,976 cases, 29,517 non-case men), we estimated an increased frequency of *HOXB13* G84E mutation carriers among men with the disease than among men with no evidence of prostate cancer in the cohort (1.87% versus 0.78% imputation-based carriers, or 1.07% versus 0.44% actual carriers adjusting

for incomplete LD). In the case-control analysis, the G84E mutation increased the risk of prostate cancer approximately two-and-a-half fold, OR = 2.52, 95% CI = 1.92–3.31, $p = 1.05\times10^{-11}$. After adjustment for incomplete LD, the OR was 3.63 (95% CI = 2.48–5.85). This finding is consistent with prior OR estimates for the G84E mutation and prostate cancer [22] and supports the accuracy of our imputation of G84E.

Fig. 4 presents the unadjusted and adjusted Kaplan-Meier curves for time-to-onset of prostate cancer for *HOXB13* G84E carriers versus non-carriers. By a log-rank test, the carriers had significantly increased age-specific risks ($p = 1.7\times10^{-12}$). For example, the LD-adjusted estimated risk of developing prostate cancer among G84E carriers was 36.7% (95% CI = 24.2%–55.4%) versus 13.6% (95% CI = 13.2%–14.2%) for non-carriers by age 72, and 64.2% (95% CI = 45.6%–93.3%) for carriers versus 24.2% (95% CI = 23.4%–25.0%) for non-carriers by age 80. Adjusting for covariates in the multivariable Cox proportional-hazards model, the estimated hazards ratio (HR) was 2.25 (95% CI = 1.78–2.84, $p = 6.5\times10^{-12}$), with an LD-adjusted HR = 3.17 (95% CI = 2.26–4.90). We then conducted two analyses to assess the impact of any potential survivor bias on these results. Including prostate cancer information in individuals up to the time at which they completed the RPGEH GERA survey (i.e., prevalent prostate cancer cases), we obtained a similar unadjusted HR of 2.48 (95% CI = 1.86–3.31, $p = 3.3\times10^{-10}$). Restricting the prostate cancer information on individuals from the time of survey onward (incident cases) and excluding any cases beforehand (i.e., prevalent cases), we derived a similar unadjusted HR of 2.14 (95% CI = 1.43–3.21, $p = 1.0\times10^{-4}$).

G84E and pleiotropy. In addition to our findings for prostate cancer, the *HOXB13* G84E mutation was associated with non-prostate cancers overall, allowing for correlation among the various cancer types (LD-adjusted OR = 1.63, 95% CI = 1.22–2.29, p = 0.00058, Table 1). According to a model of pleiotropy, we would expect individuals affected with primary cancers of more than one type to have a stronger association with the G84E mutation. This expectation was met in our data; the LD-adjusted OR for the association between the mutation and any single cancer diagnosis was 1.71 (95% CI = 1.23–2.51, p = 0.00089), while the LD-adjusted OR for being diagnosed with multiple cancer types was 3.12 (95% CI = 1. 60–6.14, p = 0.0011). Similarly, we would also expect a stronger association of the G84E mutation with prostate cancer cases that involved an additional cancer type versus those that involve only the prostate. This expectation was also met. For prostate cancer alone, the LD-adjusted OR was 3.40 (95% CI = 2.35–5.34, $p = 1.8\times10^{-12}$), while for cases diagnosed with cancer of the prostate plus an additional type, the LD-adjusted OR was 4.65 (95% CI = 2.30–9.60, $p = 3.9\times10^{-5}$).

In terms of individual cancers, we estimated an OR>1 for the *HOXB13* G84E mutation carriers in the following 12 cancer types (out of fourteen) in comparison with controls not diagnosed with any form of (non-melanoma skin) cancer: breast, non-Hodgkin's lymphoma, kidney, bladder, melanoma, endometrium, pancreas, colon, thyroid, ovary, multiple myeloma, and lung (Table 1). Note that the number of controls varied depending on whether the cancers were sex-specific; for the sex-specific cancers we only compared cases to controls of the same sex. Due to the rarity of the G84E mutation, the number of carriers among the cancer cases was limited (Table 1). Thus, while the G84E mutation was associated positively with many of the cancers individually and with all cancer typess in an overall test, the cancer-specific effects were of borderline significance or non-significant (Table 1). Additionally, when analyzing only subjects that were age > 55, results were generally similar (84% of our analytic cohort is age > 55); when analyzing the subgroup with age ≤ 55, results were generally not statistically significant except for prostate cancer (OR = 3.0, 95% CI = 1.1–8.2) and kidney cancer (OR = 8.9, 95% CI = 2.0–38.6). However, in many cases, the power is poor because of small samples sizes.

To further evaluate the cancer-specific results, we determined the optimal statistical grouping of cancer types (see Methods section). We found the risk group to include the following

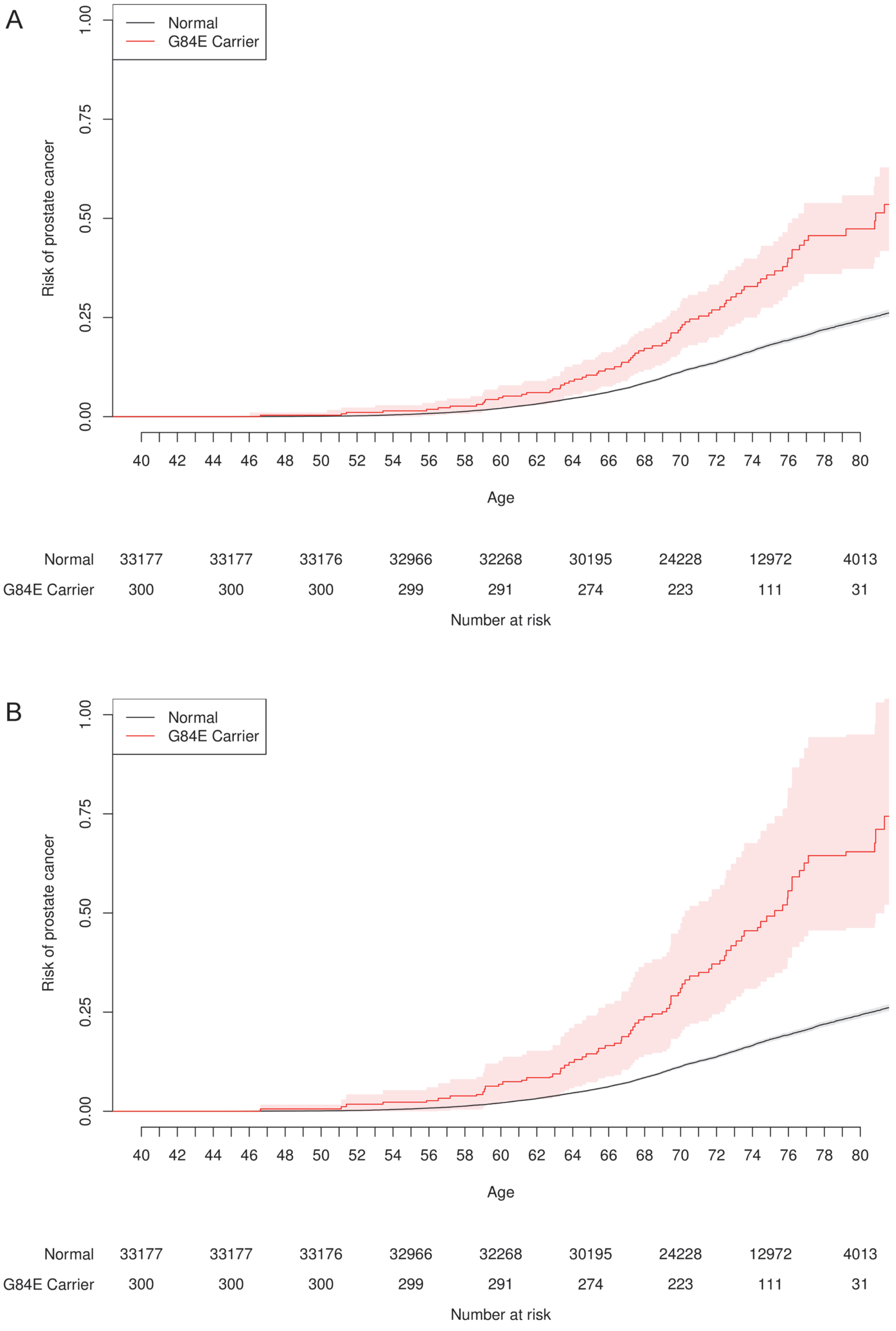
A
Normal
G84E Carrier
Risk of prostate cancer
0.00
0.25
0.50
0.75
1.00
40 42 44 46 48 50 52 54 56 58 60 62 64 66 68 70 72 74 76 78 80
Age
Normal 33177 33177 33176 32966 32268 30195 24228 12972 4013
G84E Carrier 300 300 300 299 291 274 223 111 31
Number at risk
B
Normal
G84E Carrier
Risk of prostate cancer
0.00
0.25
0.50
0.75
1.00
40 42 44 46 48 50 52 54 56 58 60 62 64 66 68 70 72 74 76 78 80
Age
Normal 33177 33177 33176 32966 32268 30195 24228 12972 4013
G84E Carrier 300 300 300 299 291 274 223 111 31
Number at risk

Figure 4. Age-specific risk of prostate cancer by *HOXB13* G84E mutation carrier status. One minus the usual Kaplan-Meier survival curve, with the probability of prostate cancer on the y-axis. The risk for G84E carriers is significantly higher than that for non-carriers. (a) Unadjusted. (b) Adjusted for incomplete LD.

doi:10.1371/journal.pgen.1004930.g004

cancers: breast, non-Hodgkin's lymphoma, kidney, bladder, melanoma, endometrium, and pancreas (combined OR = 1.50, LD-adjusted OR = 1.87, one sided p = 0.042, adjusted for multiple testing of all possible subsets).

Discussion

Although the GWAS array typed in our study cohort did not include the G84E mutation [42], we were able to impute it even though it has a low carrier frequency (0.0034 in individuals of European ancestry from the 1000 Genomes Project), given a sufficient number of mutation carriers in the reference panel used for imputation. We were able to identify a homogeneous

Table 1. Pleiotropic effect of G84E mutation on risk of cancer.

Cancer[a]	# Case carriers[b]	# Cases[b]	Frequency[c]	Odds Ratio[d] (95% CI)	Corrected Odds Ratio[e] (95% CI)	P-value[d]
Kidney	5	303	0.94%	2.32 (0.94, 5.76)	3.32 (0.89, 9.99)	0.034
Bladder	5	335	0.85%	2.05 (0.81, 5.22)	2.84 (0.70, 8.94)	0.065
Non-Hodgkin's Lymphoma	10	846	0.67%	1.81 (0.98, 3.36)	2.42 (0.96, 5.52)	0.029
Melanoma	15	1301	0.66%	1.50 (0.89, 2.55)	1.88 (0.81, 3.95)	0.066
Pancreas	2	149	0.77%	1.49 (0.28, 7.83)	1.86 (0.18, 13.70)	0.32
Breast	38	3183	0.68%	1.48 (1.04, 2.10)	1.84 (1.07, 3.16)	0.014
Endometrium	6	578	0.59%	1.44 (0.64, 3.24)	1.77 (0.49, 5.22)	0.19
Colon	11	1119	0.56%	1.42 (0.77, 2.60)	1.74 (0.65, 4.06)	0.13
Thyroid	3	217	0.79%	1.35 (0.35, 5.22)	1.61 (0.23, 8.81)	0.33
Ovary	2	198	0.58%	1.32 (0.32, 5.49)	1.56 (0.20, 9.26)	0.35
Multiple Myeloma	1	135	0.42%	1.26 (0.20, 7.92)	1.46 (0.12, 13.75)	0.40
Lung	5	667	0.43%	1.10 (0.44, 2.72)	1.18 (0.30, 4.22)	0.42
Oral	2	283	0.40%	0.98 (0.23, 4.10)	0.97 (0.14, 6.78)	NA
Lymphocytic Leukemia	1	218	0.26%	0.53 (0.05, 5.24)	0.39 (0.03, 9.06)	NA
Any[f]	123	10493	0.67%	1.36 (1.13, 1.63)	1.63 (1.22, 2.29)	5.8×10^{-4}
Any single cancer[g]	106	9532	0.63%	1.41 (1.14, 1.75)	1.72 (1.23, 2.51)	8.9×10^{-4}
Two or more cancers[h]	17	961	1.01%	2.21 (1.35, 3.61)	3.12 (1.60, 6.14)	0.0011

Estimates of the association between the G84E mutation and risk of fourteen cancer(s) in the RPGEH GERA cohort. An overall association was detected between the G84E mutation and the fourteen cancers grouped together (odds ratio = 1.36).

[a]Number of non-cancer carriers / total number of individuals without cancer (= frequency): = (430 / 54,482) × 0.57 = 0.45% for both genders[c] and = (263 / 31,965) × 0.57 = 0.47% for females.[c]

[b]The expected number of carriers according to best guess (tested using additive dosages)

[c]Adjusted for incomplete LD by multiplying the imputation carrier frequency by the r^2 estimate of 0.57.

[d]Adjusted for age and genetic ancestry. One-sided p-value (see Methods) and OR from the non-corrected analysis (with two-sided 95% CI), not adjusted for multiple comparisons. Some ORs are larger than 1.0 even though the frequencies in cases are not higher than controls because of the adjustment for covariates.

[e]Since the imputed variant is not perfectly correlated with the genotyped variant, the OR is underestimated, so we correct the estimate as for incomplete LD (see Methods).

[f]Any = any of the fourteen cancers using a meta-analysis approach that takes into account shared controls and cases.

[g]Analysis using an indicator variable for presence of any single cancer of the fourteen cancers.

[h]Analysis using an indicator variable for presence of any two or more of the fourteen cancers.

doi:10.1371/journal.pgen.1004930.t001

founder haplotype consisting of SNPs on the GWAS array that allowed us to impute the G84E mutation. We applied CART to the phased haplotypes—and introduced a novel CART-like method—to find these haplotypes and ensure that the imputation was not an artifact, and to identify the founder haplotype. This is not meant to replace imputation, but merely as an approach to examine imputation results and assure their validity. We expect that imputation will similarly be possible for many additional rare variants as sequence information from large sequencing efforts such as the UK10K project (http://www.uk10k.org) and others become publicly available for use in reference panels. Furthermore, with the expansion of reference panels, the number of copies of rare variants within them will increase. This will obviate the need for creating custom sequenced reference panels oversampled for mutation carriers as was the case here, and hence also avoid the biases in LD estimates that occur with such oversampling. However, special care in analysis will have to be taken, as rare variants would often not meet standard QC filtering metrics.

In our data example, we obtained confirmatory evidence of the association of the *HOXB13* G84E mutation with prostate cancer, and provided age-specific risks for developing prostate cancer for mutation carriers in a large, prospective cohort. We also provided evidence that the G84E mutation exhibits a pleiotropic effect on numerous other cancers, though sample sizes made it difficult to determine precisely which cancers are involved. Consistent with the hypothesis of pleiotropy, we also provided suggestive evidence that the mutation exhibits a stronger association in individuals with multiple cancers, both involving prostate cancer and independent of prostate cancer. Multiple cancers in the same individual will most often arise independently and may reflect pleiotropic events, though in some cases may be due to metastasis. A shared genetic basis among cancers may be supported by *HOXB13*'s role in embryonic development and body patterning [20,43,44]. *HOXB13* is particularly expressed in the prostate [20], where it physically interacts with the androgen receptor, which is important for growth and regulation of differentiation in normal cell biology. Thus, *HOXB13* may impact the carcinogenic process via its action on growth and development. More work is needed to examine the biological mechanisms and effects that the mutation has on the function of the *HOXB13* gene. Two key factors made this investigation possible: a very large genotyped cohort with information on multiple cancers and our ability to impute the G84E mutation using a custom reference panel.

Our LD-adjusted OR estimate of 3.63 (and HR of 3.17) for the overall association between the G84E mutation and prostate cancer is slightly smaller than that reported previously (meta-analysis OR = 4.51) [22,27], although the latter is well within the 95% confidence interval of our OR estimate (2.48–5.85). Our overall estimate may also reflect the older average age of the RPGEH GERA cohort, since we did observe a stronger association when restricting to younger (age <55) men. Due to the small number of G84E carriers, we were underpowered to detect associations for non-prostate cancers (power for prostate > 99%, breast 20%, and others < 10%, using the parameters in Table 1 with alpha 0.0036 (0.05/14)). Nevertheless, our pleiotropy analysis was able to borrow strength by combining across cancers and looking at individuals with multiple cancers, all of which supported an association with cancer more broadly, albeit with lower risks than associated with prostate cancer. On the other hand, our estimated LD-adjusted OR for multiple cancers excluding prostate (3.12) did approach the OR for prostate cancer alone (3.40). Another potential issue is the inclusion of prevalent cancer cases: these individuals may have less aggressive disease and survive longer, making them more likely to become members of the RPGEH GERA cohort. If individuals with prevalent cancer are less likely to carry the HOXB13 G84E mutation and were included preferentially due to survival bias, our results would underestimate any true associations. Nevertheless, this potential bias is minimized for prostate cancer since men with this disease most commonly die from other factors. The results for prevalent and incident prostate cancer cases gave similar results, suggesting that there was no or very little survivor bias.

In contrast with our work, a previous study reported difficulty in imputing the G84E variant using a large custom reference panel [37]. This likely reflects differences in genotyping arrays, imputation, or r^2 estimation approaches. Saunders et al. [37] used multi-panel imputation with Impute2 v2.3.0 [45] to create a large reference panel comprised of: 5500 prostate cancer cases and 4923 controls typed on their custom iCOGS cancer array plus G84E; 677 cases typed on the OMNI 2.5 array; and 1000 Genomes subjects. With this reference panel, they imputed the HOXB13 G84E into 14,940 prostate cancer cases and 16,546 controls that were typed on the iCOGS array; however, they reported inadequate imputation quality [37]. We investigated whether this was due to the genotyping array by restricting our genotypes to the SNPs on the iCOGS array and repeating our imputation approach. Approximately 50% of the SNPs on our array in this interval were also on the iCOGS array, and represented approximately 10% of all SNPs on their array in this interval. Limiting our imputation to those SNPs that overlapped between our array and the iCOGS array, we still obtained ${r_{info}}^2 = 0.74$ and $r^2 = 0.40$ (95% CI = 0.16–0.64) with the genotyped data. Because our imputation was reasonably successful with a relatively small subset of SNPs on their array, we suspect that there may have been further explanations for their low r^2 value, possibly including differences in the approaches taken to impute the variant or potential difficulty in accurately estimating r^2.

Our ability to impute the HOXB13 G84E mutation and calculate the age-specific risk of prostate cancer for carriers, along with evidence for association with a number of other cancers, highlights the value of combining sequence data with a large cohort of genotyped individuals to assess the impact of rare variants on multiple diseases.

Material and Methods

Human subjects protection

The study was approved by the Kaiser Permanente and University of California Institutional Review Boards.

Constructing a reference panel for imputation

The G84E mutation is present in only two of the 1092 individuals in the 1000 Genomes Project March 2012 interim release dataset: one British (GBR) and one Finnish (FIN), and is not present in other race/ethnicity groups. We estimated the proportion of European ancestry in 1000 Genomes using Admixture v1.23 [46] to be 581.2 individuals. Thus we estimate a carrier frequency of 0.0034 (MAF 0.0017) among individuals of European ancestry.

These numbers were insufficient to create a reference panel for imputation, so we added to this reference population a group of 93 individuals of European descent, 22 of whom were known carriers of the G84E mutation (by sequencing), to create an enriched reference panel. To ascertain G84E status, the DNA of those 93 individuals was amplified by PCR and sequenced (Genewiz, La Jolla, CA), as described previously [30]. These 93 individuals were also genotyped on the custom Affymetrix Axiom EUR array optimized for individuals of European descent as described in [42,47], the same array on which the non-Hispanic White RPGEH GERA individuals were genotyped. The individuals were combined together with all of the 1000 Genomes Project data on the overlapping SNPs, and then phased with Shape-IT v2.r727 [48].

Participants in the target population

The RPGEH GERA cohort is comprised of 103,006 ethnically diverse Kaiser Permanente Northern California (KPNC) health plan members (7.7% Asian, 3.4% African American, 7.2% Latino, and 81.0% non-Hispanic White; 42% male) who were genotyped at over 674,000 SNPs

on four race/ethnicity specific arrays [42,47]. Each cohort member also completed a baseline health survey that included a list of self-reported medical conditions and lifestyle factors. These individuals were an average age of 62.9 years at specimen collection (in 2008–2009), have been members of KPNC for 23.5 years on average, and have comprehensive Electronic Health Records (EHR), tumor registry information, and other data available (e.g., cancer diagnosis). For this analysis, we focused on the non-Hispanic White individuals, as the G84E mutation appears to be of European origin. These individuals were genotyped on the same array as the extra 93 individuals who were used to create the enriched reference panel. The GERA cohort constitutes a subset of the entire RPGEH sample.

Pre-imputation quality control

Algorithms for genotype calling for the Affymetrix Axiom arrays and QC measures for the RPGEH GERA cohort have been described elsewhere (dbGaP, phs000674.v1.p1). In addition, we applied stricter QC measures for this analysis; in particular, SNPs were removed if they had an overall call rate <0.95, or a Hardy-Weinberg p-value $<2.6\times10^{-4}$ (a Bonferroni correction of 0.05/186, for the 186 SNPs +/− 0.5MB G84E on the EUR array) leaving a total of 170 SNPs passing QC.

Imputation and confirmation

Imputation was performed by pre-phasing the RPGEH GERA cohort genotypes on each of the arrays with Shape-IT v2.r727 [48], using the genotypes of all RPGEH GERA individuals, including first-degree relatives modeled as such to improve phasing. The G84E variant was then imputed using the enriched reference panel with Impute2 v2.3.0 [45,49,50]. The estimated r_{info}^2 metric we provide is the “info” metric from Impute2, which is an estimate of the correlation of the imputed genotype to the true genotype [51]. The expected frequencies/counts from summing the additive dosages are typically given in the text and are additionally adjusted for incomplete LD (we multiplied the imputation carrier frequency by the r^2 estimate of 0.57); in some presentations we used the most likely genotype/best guess genotypes, in which cases it is explicitly stated as such. All regression analyses described below used the additive dosages.

To further confirm the imputation, we first employed a CART method to identify the founder haplotype in the enriched reference panel, and predict G84E in the RPGEH GERA cohort using the tree model. We restricted our search space to +/− 3 crossovers (recombination distance), which spans from 46.684–46.908 Mb on chromosome 17q21.32, and leaves 57 SNPs to reconstruct the haplotype. The CART method uses the pre-phased haplotypes, and predicts the G84E mutation using standard CART methods based on each estimated haplotype [40] with the R package rpart [52]. We obtained the probability of the G84E mutation from the classification in the terminal nodes. To further confirm that imputation of the mutation was not relying on a single or small number of SNPs, we removed the SNPs selected by the CART approach, and repeated the process twice, in the second round removing SNPs identified in the first round, and in the third round removing the SNPs identified in the first two rounds.

Our final computational confirmation was with the software PLINK v1.07 [38], used to identify haplotype proxies and compute a multi-marker correlation with the G84E mutation using our enriched reference panel and the non-Hispanic White individuals in the 1000 Genomes Project. As for the CART analysis, we repeated this analysis by masking the SNPs used in the original multi-marker correlation and re-computing the multi-marker correlation to show it also does not rely on any single or small number of SNPs.

As a final confirmation, we genotyped the G84E mutation on a subset of 1,673 RPGEH GERA individuals along with an additional 1,789 men of White race/ethnicity from the CMHS

[39], using a custom Affymetrix Axiom microarray. QC criteria were similar to those described earlier; genotyping calling for the G84E allele was performed using all individuals together. We repeated the imputation by combining the non-Hispanic white RPGEH GERA individuals with the additional 1,789 men from the CMHS (who were also genotyped on the same Axiom EUR array as the RPGEH GERA individuals) to create a larger set of individuals to empirically test the accuracy of imputation.

Leave-one-out cross validation estimate of r^2

We also estimated the r^2 of our reference panel via leave-one-out cross validation (LOOCV), imputing each individual using the genotype information of all other individuals. In our reference panel, we have enriched for *HOXB13* carriers, leading to an increased frequency compared to the general population.

Comparison of imputed to genotyped mutation carriers

To illustrate why there is a discrepancy between the ${r_{info}}^2$ estimate from the enriched reference panel and the correlation estimate from the genotype data, we downward adjusted the original r^2 estimate to account for the over-sampling of mutation carriers. Recall that we estimated a carrier frequency of 0.0034 in individuals of European ancestry in the 1000 Genomes Project data; using this same proportion for the additional 93 individuals in the reference sample would give an expected 0.32 carriers (i.e., the additional 93 individuals were enriched 69-fold). Thus, for the enriched reference panel (1000 Genomes data plus 93 extra individuals), we would expect 2 + 0.32 = 2.32 mutation carriers with the founder haplotype and 1 person with the founder haplotype but without the mutation (as was found when we reversed the process and incorrectly identified a carrier). This gives an estimated r^2 of approximately 2.32/3.32 = 0.70.

Confidence intervals for r^2 estimates

Confidence intervals for estimates were given either by using the adjusted bootstrap percentile method [53] for correlations with the genotyped subset, and for the other more computationally demanding estimates via the bootstrap percentile.

Application to a large cohort

Removal of first-degree relatives. For the phenotype analysis described here, relatives were randomly removed such that no first-degree relationships remained except in cases in which there were multiple relationships, where removal was based on maximizing the remaining number of unrelated individuals (e.g., the two parents in trios). This resulted in a total of 78,948 unrelated non-Hispanic White individuals with valid genotype data, before exclusions based on phenotype.

Phenotype. Prostate cancer cases were identified from the Kaiser Permanente Northern California Cancer Registry (KPNCCR) and clinical I data through the end of 2012. The KPNCCR captures data on all cancer cases (except non-melanoma skin cancer) newly-diagnosed or treated at KPNC facilities. Data in the KPNCCR conform to standards of the North American Association of Central Cancer Registries and the National Cancer Instit'te's Surveillance, Epidemiology and End Results (SEER) Program. For prostate cancer, non-case individuals were all men who had not progressed to prostate cancer.

To examine the pleiotropic effect of the G84E mutation, other cancer cases were identified from the KPNCCR. Controls were individuals without any current or previous cancer diagnosis (additionally excluding individuals with ICD-9 diagnoses of cancer or self-reported history of cancer from the survey), except for non-melanoma skin cancer. Including all men for

estimating age-specific risk of prostate cancer (i.e., not excluding them for other phenotypic exclusions, but still removing first-degree relatives), and these exclusions for cases and controls, a total of 74,625 individuals were used in the cancer association analyses (this is the total number of individuals used across all tests, the numbers of individuals vary for each analysis).

Analysis and covariate adjustment. The G84E mutation was tested for association with prostate and other cancers with a logistic regression model using the imputed probabilities to construct additive dosages to account for the uncertainty of imputation. This has been shown to work well in practice [54], although not yet for such a rare mutation. However, because of the rarity of the mutation, in this case dosage is very highly correlated with predicted carrier status since very few homozygotes are expected. For the CART method, we used the probability of the mutation G84E from the classification in the terminal nodes. All regression models were adjusted for age and genetic ancestry (described below).

We also conducted a survival analysis of the time-to-onset of prostate cancer in order to estimate the age-specific risk for carriers versus non-carriers of the mutation. We used age at diagnosis for the affected men, and censored unaffected men at their age at time of latest observation. We calculated Kaplan-Meier lifetime risk estimates for G84E carriers versus non-carriers and evaluated their difference with a log-rank test, and also conducted a multivariable Cox proportional-hazards model adjusting for genetic ancestry.

To adjust for genetic ancestry/population stratification, principal components (PC) analysis was performed on a set of 20,000 non-Hispanic White race/ethnicity individuals, with the remaining non-Hispanic White race/ethnicity individuals projected into the same space via Eigensoft v4.2 [55]. The top 10 eigenvectors were included in the logistic regression model. We were also interested in evaluating the geographic distribution of the G84E mutation. To do so, we created a smoothed estimate of the carrier probability for each individual by calculating the carrier frequency among the 2,000 closest individuals to them (in Euclidean genetic distance) and then plotted carrier probabilities as a function of PC scores.

Each cancer phenotype was initially modeled separately via logistic regression. To evaluate all cancers together as a group, allowing for correlation in the occurrence of the different cancers, and to assess which group of cancers appeared to jointly exhibit pleiotropic effects due to G84E, we implemented a previously described subset-based approach [56]. Briefly, the approach computes a statistic for each subset of cancers, calculated as a weighted sum of the univariate case-control analysis Z statistics for each cancer, taking into account shared controls and shared cases. It then chooses the maximum of these subsets (which we report here), and computes a combined odds ratio and p-value adjusted for taking the maximum of all possible subsets. When analyzing all the cancers together (excluding prostate), we implemented this approach with just one subset, so it was effectively just a meta-analysis taking into account shared controls and cases. Then we used the method testing all possible subsets to determine the optimal statistical grouping of cancer types. Finally, for the other tests of any single non-prostate cancer, 2 or more cancers, and prostate cancer and another cancer, we tested an indicator variable of this condition. For all cancer phenotype tests, we conducted a one-sided test as we are interested in determining whether the mutation increases the risk of cancer, and thus reported a one-sided p-value; all confidence intervals are two-sided.

Adjusting risk estimates for the effect of incomplete linkage disequilibrium. Because our imputation of the *HOXB13* mutation is imperfect due to incomplete linkage disequilibrium, there is some misclassification of carrier status. Such misclassification leads to an underestimate of the true effect of the mutation on prostate and other cancers. To address this, we used the following approach to adjust effect estimates, using the r^2 computed from the mutation genotyped subset ($r^2 = 0.57$, 95% CI = 0.37–0.77). In our results we always provide the unadjusted results, and in relevant situations adjusted estimates accounting for incomplete LD.

Let Z, X, and Y be indicator variables for affection status, mutation carrier status, and haplotype carrier status, respectively. Let a = P(Z = 1|X = 1) = probability affected if mutation carrier; b = P(Z = 1|X = 0) = probability affected if not a mutation carrier; f = P(Z = 1|Y = 1) = probability affected if haplotype carrier; g = P(Z = 1|Y = 0) = probability affected if not haplotype carrier; v = a/b = relative risk for mutation carriers (not observed); and w = f/g = relative risk for haplotype carriers (observed). The calculation is greatly simplified by assuming that the G84E mutation occurred on a single ancestral haplotype, and therefore that haplotype is perfectly specific but imperfectly sensitive for carrying the mutation. This allows us to assume that P (X = 1|Y = 0) = 0. Further assume that a proportion t of haplotype carriers also carry the G84E mutation, namely P(X = 1|Y = 1) = t. Then f = P(Z = 1|Y = 1) = P(X = 1|Y = 1)P(Z = 1|X = 1)+ P(X = 0|Y = 1)P(Z = 1|X = 0) = at + b(1-t). Similarly, g = P(Z = 1|Y = 0) = P(X = 1|Y = 0)P(Z = 1|X = 1)+P(X = 0|Y = 0)P(Z = 1|X = 0) = b. As a consequence, w = f/g = [at+b(1-t)]/b = t(a/b) + 1-t = tv+1-t. Solving for v in terms of w, we obtain v = 1 + (w-1)/t. The value for v gives the adjusted relative risk for mutation carriers. The values for a and b give the adjusted survival curve estimates for carriers and non-carriers. Because non-haplotype carriers are not mutation carriers, the risk for non-mutation carriers will be the same as the risk for non-haplotype carriers. To adjust the confidence intervals, we computed nonparametric bootstrapped confidence intervals (10^8 iterations, except 10^5 iterations for the time-to-onset plots), drawing from a normal distribution with mean and standard error of the coefficient estimate, and inflating by the r^2 from drawing from a normal distribution with mean and standard error from the estimate for r^2 from the genotyped subset.

Acknowledgments

We are grateful to the Kaiser Permanente Northern California members who have generously agreed to participate in the Kaiser Permanente Research Program on Genes, Environment and Health and the California Men's Health Study.

Author Contributions

Conceived and designed the experiments: TJH LCS SKVDE NR JSW. Analyzed the data: TJH LCS LS JL MNK SKVDE NR JSW. Wrote the paper: TJH LCS LS SKVDE NR JSW. Critical revision of the manuscript: EJ LAH MNK MMA YB DC LHK CPQ CS.

References

1. Dickson SP, Wang K, Krantz I, Hakonarson H, Goldstein DB (2010) Rare Variants Create Synthetic Genome-Wide Associations. PLoS Biol 8: e1000294. PMID: 20126254
2. Sham PC, Purcell SM (2014) Statistical power and significance testing in large-scale genetic studies. Nat Rev Genet 15: 335–346. PMID: 24739678
3. Cirulli ET, Goldstein DB (2010) Uncovering the roles of rare variants in common disease through whole-genome sequencing. Nat Rev Genet 11: 415–425. doi: 10.1038/nrg2779 PMID: 20479773
4. Welter D, MacArthur J, Morales J, Burdett T, Hall P, et al. (2014) The NHGRI GWAS Catalog, a curated resource of SNP-trait associations. Nucleic Acids Res 42: D1001–D1006. doi: 10.1093/nar/gkt1229 PMID: 24316577
5. Zheng H-F, Ladouceur M, Greenwood CMT, Richards JB (2012) Effect of Genome-Wide Genotyping and Reference Panels on Rare Variants Imputation. Journal of Genetics and Genomics 39: 545–550. PMID: 23089364
6. Auer PL, Johnsen JM, Johnson AD, Logsdon BA, Lange LA, et al. (2012) Imputation of Exome Sequence Variants into Population- Based Samples and Blood-Cell-Trait-Associated Loci in African Americans: NHLBI GO Exome Sequencing Project. The American Journal of Human Genetics 91: 794–808. doi: 10.1016/j.ajhg.2012.08.031 PMID: 23103231

7. Duan Q, Liu EY, Auer PL, Zhang G, Lange EM, et al. (2013) Imputation of coding variants in African Americans: better performance using data from the exome sequencing project. Bioinformatics 29: 2744–2749. doi: 10.1093/bioinformatics/btt477 PMID: 23956302

8. Holm H, Gudbjartsson DF, Sulem P, Masson G, Helgadottir HT, et al. (2011) A rare variant in MYH6 is associated with high risk of sick sinus syndrome. Nature Genetics 43: 316–320. doi: 10.1038/ng.781 PMID: 21378987

9. Jewett EM, Zawistowski M, Rosenberg NA, Zöllner S (2012) A Coalescent Model for Genotype Imputation. Genetics 191: 1239–1255. PMID: 22595242

10. Joshi PK, Prendergast J, Fraser RM, Huffman JE, Vitart V, et al. (2013) Local Exome Sequences Facilitate Imputation of Less Common Variants and Increase Power of Genome Wide Association Studies. PLoS ONE 8: e68604. doi: 10.1371/journal.pone.0068604 PMID: 23874685

11. Jostins L, Morley KI, Barrett JC (2011) Imputation of low-frequency variants using the HapMap3 benefits from large, diverse reference sets. European Journal of Human Genetics 19: 662–666. doi: 10.1038/ejhg.2011.10 PMID: 21364697

12. Li L, Li Y, Browning SR, Browning BL, Slater AJ, et al. (2011) Performance of Genotype Imputation for Rare Variants Identified in Exons and Flanking Regions of Genes. PLoS ONE 6: e24945. doi: 10.1371/journal.pone.0024945 PMID: 21949800

13. Liu EY, Buyske S, Aragaki AK, Peters U, Boerwinkle E, et al. (2012) Genotype Imputation of MetabochipSNPs Using a Study-Specific Reference Panel of ∼4,000 Haplotypes in African Americans From the Women's Health Initiative. Genetic Epidemiology 36: 107–117. doi: 10.1002/gepi.21603 PMID: 22851474

14. Mägi R, Asimit JL, Day-Williams AG, Zeggini E, Morris AP (2012) Genome-Wide Association Analysis of Imputed Rare Variants: Application to Seven Common Complex Diseases. Genetic Epidemiology 36: 785–796. doi: 10.1002/gepi.21675 PMID: 22951892

15. Sung YJ, Wang L, Rankinen T, Bouchard C, Rao DC (2012) Performance of Genotype Imputations Using Data from the 1000 Genomes Project. Human Heredity 73: 18–25. PMID: 22212296

16. Wood AR, Perry JRB, Tanaka T, Hernandez DG, Zheng H-F, et al. (2013) Imputation of Variants from the 1000 Genomes Project Modestly Improves Known Associations and Can Identify Low-frequency Variant—Phenotype Associations Undetected by HapMap Based Imputation. PLoS ONE 8: e64343. doi: 10.1371/journal.pone.0064343 PMID: 23696881

17. Akbari MR, Trachtenberg J, Lee J, Tam S, Bristow R, et al. (2012) Association Between Germline HOXB13 G84E Mutation and Risk of Prostate Cancer. JNCI J Natl Cancer Inst 104: 1260–1262. PMID: 22781434

18. Breyer JP, Avritt TG, McReynolds KM, Dupont WD, Smith JR (2012) Confirmation of the HOXB13 G84E Germline Mutation in Familial Prostate Cancer. Cancer Epidemiol Biomarkers Prev 21: 1348–1353. PMID: 22714738

19. Chen Z, Greenwood C, Isaacs WB, Foulkes WD, Sun J, et al. (2013) The G84E mutation of HOXB13 is associated with increased risk for prostate cancer: results from the REDUCE trial. Carcinogenesis 34: 1260–1264. doi: 10.1093/carcin/bgt055 PMID: 23393222

20. Ewing CM, Ray AM, Lange EM, Zuhlke KA, Robbins CM, et al. (2012) Germline mutations in HOXB13 and prostate-cancer risk. New England Journal of Medicine 366: 141–149. PMID: 22236224

21. Gudmundsson J, Sulem P, Gudbjartsson DF, Masson G, Agnarsson BA, et al. (2012) A study based on whole-genome sequencing yields a rare variant at 8q24 associated with prostate cancer. Nature Genetics 44: 1326–9. doi: 10.1038/ng.2437 PMID: 23104005

22. Huang H, Cai B (2014) G84E mutation in HOXB13 is firmly associated with prostate cancer risk: a meta-analysis. Tumor Biol 35: 1177–1182. doi: 10.1007/s13277-013-1157-5 PMID: 24026887

23. International Consortium for Prostate Cancer Genetics, Xu J, Lange EM, Lu L, Zheng SL, et al. (2012) HOXB13 is a susceptibility gene for prostate cancer: results from the International Consortium for Prostate Cancer Genetics (ICPCG). Human Genetics.

24. Karlsson R, Aly M, Clements M, Zheng L, Adolfsson J, et al. (2014) A Population-based Assessment of Germline HOXB13 G84E Mutation and Prostate Cancer Risk. European Urology 65: 169–176. PMID: 22841674

25. Klu niak W, Wokołorczyk D, Kashyap A, Jakubowska A, Gronwald J, et al. (2013) The G84E mutation in the HOXB13 gene is associated with an increased risk of prostate cancer in Poland. The Prostate 73: 542–548. doi: 10.1002/pros.22594 PMID: 23334858

26. Laitinen VH, Wahlfors T, Saaristo L, Rantapero T, Pelttari LM, et al. (2013) HOXB13 G84E Mutation in Finland: Population-Based Analysis of Prostate, Breast, and Colorectal Cancer Risk. Cancer Epidemiol Biomarkers Prev 22: 452–460. doi: 10.1158/1055-9965.EPI-12-1000-T PMID: 23292082

27. MacInnis RJ, Severi G, Baglietto L, Dowty JG, Jenkins MA, et al. (2013) Population-Based Estimate of Prostate Cancer Risk for Carriers of the HOXB13 Missense Mutation G84E. PLoS ONE 8: e54727. doi: 10.1371/journal.pone.0054727 PMID: 23457453

28. Shang Z, Zhu S, Zhang H, Li L, Niu Y (2013) Germline Homeobox B13 (HOXB13) G84E Mutation and Prostate Cancer Risk in European Descendants: A Meta-analysis of 24 213 Cases and 73 631 Controls. European Urology 64: 173–176. doi: 10.1016/j.eururo.2013.03.007 PMID: 23518396

29. Stott-Miller M, Karyadi DM, Smith T, Kwon EM, Kolb S, et al. (2013) HOXB13 mutations in a population-based, case-control study of prostate cancer. The Prostate 73: 634–641. doi: 10.1002/pros.22604 PMID: 23129385

30. Witte JS, Mefford J, Plummer SJ, Liu J, Cheng I, et al. (2013) HOXB13 Mutation and Prostate Cancer: Studies of Siblings and Aggressive Disease. Cancer Epidemiol Biomarkers Prev 22: 675–680. doi: 10.1158/1055-9965.EPI-12-1154 PMID: 23396964

31. Miao J, Wang Z, Provencher H, Muir B, Dahiya S, et al. (2007) HOXB13 promotes ovarian cancer progression. PNAS 104: 17093–17098. PMID: 17942676

32. Marra L, Cantile M, Scognamiglio G, Perdonà S, La Mantia E, et al. (2013) Deregulation of HOX B13 expression in urinary bladder cancer progression. Curr Med Chem 20: 833–839. PMID: 23276138

33. Sabatino R, Cantile M, Aquino G, Scognamiglio G, Madonna C, et al. (2013) PP076: HOX B13 and HOX C13 expression in oral squamous cell carcinoma: A tissue microarray based immunohistochemical study. Oral Oncology 49, Supplement 1: S120.

34. Jerevall P-L, Jansson A, Fornander T, Skoog L, Nordenskjöld B, et al. (2010) Predictive relevance of HOXB13 protein expression for tamoxifen benefit in breast cancer. Breast Cancer Res 12: R53. doi: 10.1186/bcr2612 PMID: 20649975

35. Akbari MR, Anderson LN, Buchanan DD, Clendenning M, Jenkins MA, et al. (2013) Germline HOXB13 p.Gly84Glu mutation and risk of colorectal cancer. Cancer Epidemiology 37: 424–427. doi: 10.1016/j.canep.2013.03.003 PMID: 23541221

36. Alanee S, Couch F, Offit K (2012) Association of a HOXB13 Variant with Breast Cancer. New England Journal of Medicine 367: 480–481. doi: 10.1056/NEJMc1205138 PMID: 22853031

37. Saunders EJ, Dadaev T, Leongamornlert DA, Jugurnauth-Little S, Tymrakiewicz M, et al. (2014) Fine-Mapping the HOXB Region Detects Common Variants Tagging a Rare Coding Allele: Evidence for Synthetic Association in Prostate Cancer. PLoS Genet 10: e1004129. doi: 10.1371/journal.pgen.1004129 PMID: 24550738

38. Purcell S, Neale B, Todd-Brown K, Thomas L, Ferreira MAR, et al. (2007) PLINK: A Tool Set for Whole-Genome Association and Population-Based Linkage Analyses. The American Journal of Human Genetics 81: 559–575. PMID: 17701901

39. Enger SM, Van Den Eeden SK, Sternfeld B, Loo RK, Quesenberry CP, et al. (2006) California Men's Health Study (CMHS): a multiethnic cohort in a managed care setting. BMC Public Health 6: 172. PMID: 16813653

40. Breiman L, Friedman JH, Olshen RA, Stone CJ (1984) Classification and regression trees. Wadsworth International Group. 376 p.

41. Li JZ, Absher DM, Tang H, Southwick AM, Casto AM, et al. (2008) Worldwide Human Relationships Inferred from Genome-Wide Patterns of Variation. Science 319: 1100–1104. doi: 10.1126/science.1153717 PMID: 18292342

42. Hoffmann TJ, Kvale MN, Hesselson SE, Zhan Y, Aquino C, et al. (2011) Next generation genome-wide association tool: Design and coverage of a high-throughput European-optimized SNP array. Genomics 98: 79–89. doi: 10.1016/j.ygeno.2011.04.005 PMID: 21565264

43. Jung C, Kim R-S, Zhang H-J, Lee S-J, Jeng M-H (2004) HOXB13 Induces Growth Suppression of Prostate Cancer Cells as a Repressor of Hormone-Activated Androgen Receptor Signaling. Cancer Res 64: 9185–9192. PMID: 15604291

44. Norris JD, Chang C-Y, Wittmann BM, Kunder RS, Cui H, et al. (2009) The Homeodomain Protein HOXB13 Regulates the Cellular Response to Androgens. Molecular Cell 36: 405–416. doi: 10.1016/j.molcel.2009.10.020 PMID: 19917249

45. Howie BN, Donnelly P, Marchini J (2009) A Flexible and Accurate Genotype Imputation Method for the Next Generation of Genome-Wide Association Studies. PLoS Genet 5: e1000529. doi: 10.1371/journal.pgen.1000529 PMID: 19543373

46. Alexander DH, Novembre J, Lange K (2009) Fast model-based estimation of ancestry in unrelated individuals. Genome Res 19: 1655–1664. doi: 10.1101/gr.094052.109 PMID: 19648217

47. Hoffmann TJ, Zhan Y, Kvale MN, Hesselson SE, Gollub J, et al. (2011) Design and coverage of high throughput genotyping arrays optimized for individuals of East Asian, African American, and Latino

race/ethnicity using imputation and a novel hybrid SNP selection algorithm. Genomics 98: 422–430. doi: 10.1016/j.ygeno.2011.08.007 PMID: 21903159

48. Delaneau O, Marchini J, Zagury J-F (2012) A linear complexity phasing method for thousands of genomes. Nature Methods 9: 179–181. doi: 10.1038/nmeth.1785 PMID: 22138821
49. Howie B, Marchini J, Stephens M (2011) Genotype Imputation with Thousands of Genomes. G3 1: 457–470. PMID: 22384356
50. Howie B, Fuchsberger C, Stephens M, Marchini J, Abecasis GR (2012) Fast and accurate genotype imputation in genome-wide association studies through pre-phasing. Nature Genetics 44: 955–959. doi: 10.1038/ng.2354 PMID: 22820512
51. Marchini J, Howie B (2010) Genotype imputation for genome-wide association studies. Nat Rev Genet 11: 499–511. PMID: 20517342
52. R Core Team (2012) R: A language and environment for statistical computing. Available: http://www.R-project.org.
53. Efron B (1987) Better Bootstrap Confidence Intervals. Journal of the American Statistical Association 82: 171–185.
54. Huang L, Li Y, Singleton AB, Hardy JA, Abecasis G, et al. (2009) Genotype-Imputation Accuracy across Worldwide Human Populations. The American Journal of Human Genetics 84: 235–250. doi: 10.1016/j.ajhg.2009.01.013 PMID: 19215730
55. Price AL, Patterson NJ, Plenge RM, Weinblatt ME, Shadick NA, et al. (2006) Principal components analysis corrects for stratification in genome-wide association studies. Nature Genetics 38: 904–909. PMID: 16862161
56. Bhattacharjee S, Rajaraman P, Jacobs KB, Wheeler WA, Melin BS, et al. (2012) A Subset-Based Approach Improves Power and Interpretation for the Combined Analysis of Genetic Association Studies of Heterogeneous Traits. The American Journal of Human Genetics 90: 821–835. doi: 10.1016/j.ajhg.2012.03.015 PMID: 22560090
